# Round the Hospitals

**Published:** 1919-02-22

**Authors:** 


### ROUND THE HOSPITALS.

### WILL THE COLLEGE BE CAPTURED?

Every member of the College should make it her business to master what happened at a meeting of a Conference on the question of nurses' hours which was held under the auspices of the National Council of Women at 39 Victoria Street, S.W., on the 18th inst. The speakers included Major-General Cuthbert Wallace, C.B., of St. Thomas's Hospital, the Chairman of the National Council, Mrs. Ogilvie Gordon, Mrs. Bedford Fenwick, and others. Some thirty people were present, including matrons and nurses. The Chairman moved that the whole

**ROUND THE HOSPITALS—(*continued*).**

question of nurses' hours be referred to a Committee, which would report later to a subsequent meeting, which was carried *nem. con.*, the Committee to consist of three representatives from the College of Nursing, three members of the Royal British Nurses' Association, and Poor-Law nurses. Mrs. Bedford Fenwick, in answer to the Chairman, stated that "all the organised nurses' societies were now affiliated with the Royal British Nurses' Association, which would represent them." These societies, on which the Stage Army has rested for so many years, are therefore apparently *non est.* The Chairman next referred to a letter from Miss Rundle, stating that the College of Nursing had appointed a Committee to deal with the question of nurses' salaries. She asked the meeting whether they did not consider the question of salaries to be closely connected with the hours of work, and that the same body had better deal with both. Several present agreed to this, and the Chairman finally informally asked the meeting to assent to the question of nurses' salaries being included in the scope of the Committee just appointed by the National Council. It was obviously unnecessary, she added, for two Committees to be at work on such closely allied subjects.

**A TRAP FOR THE COLLEGE.**

No protest was raised to this. It all sounded very fair and reasonable; but in practice it amounts to this, that the one piece of work undertaken by the College, upon which the whole Nursing profession is anxiously awaiting its report, was thus formally seized, bag and baggage, for the new Committee just appointed. The discourtesy of this act on the part of the so-called Conference no one seemed to realise, although no sanction or reference of any kind on the part of the College was produced or referred to, nor was the fact recognised that the absorption of the College Committee on salaries, etc., could be none of the business of the meeting of the National Council of Women and those present. We await further developments, contenting ourselves with the comment that if the Council of the College of Nursing quietly suffers the capture of its Committee and the work it is responsible for, "Ierne's" criticisms will be more than justified and the future of the College imperilled. Nurses will please observe that in plain English the College has fallen into a trap, and its progress must necessarily be stayed, if not prevented altogether, unless the Council of the College takes immediate action. Meanwhile we are glad to learn that the Committee set up by the College to consider and report upon the conditions of employment of nurses and the salaries paid to them is now in session.

---

No part of the work achieved by Miss Luckes is more worthy of remembrance than her annual letters to her sisters and nurses. As we write we have before us a long series of these letters, stretching over twenty-two years. The one written in 1894 records that the nursing staff then numbered 230 and the private staff forty-five. It closes with the motto "Not failure but low aim, is crime." The one written in 1916 was the last of the long series. The hospital staff had risen to 570, the private staff to 229. Though then only partially recovered from severe illness, Miss Luckes wrote with all her usual felicity, bidding her nurses remember in the midst of the world sorrow that "character soul can only be gained by self-surrender; and self-surrender comes not of knowledge, but of love." Between these two lie a wealth of wisdom, of faithful counsels, and inspiring thought. We trust that among the memorials contemplated at the London of their beloved matron the publication of these letters, which constitute a history of the institution no less than a most valuable source of education for its future nurses, will not be omitted.

---

The King and Queen have sent the following telegram to Lord Knutsford, chairman of the London Hospital: "The Queen and I are grieved to hear of the death of Miss Luckes, and condole with you and all connected with the London Hospital in the irreparable loss of one whose life has been devoted to this noble and unbounded work of mercy."

---

Good news from Manchester. The Rt. Hon. Lord Cawley of Prestwich, President of Ancoats Hospital, Manchester, has intimated his intention of forwarding to the treasurer of the hospital £10,000 of War Scrip for the purpose of building a home for the Ancoats Hospital nurses. The home is to be built in memory of his three sons killed in the war, the youngest of whom was a member of the Committee of Management of this hospital. It is a very touching and beautiful instinct which is leading mourners to perceive that the memorial of all others which those who have given their lives for us would prefer is a living one "associated with nurses."

---

It is good news to be able to announce that at Charing Cross Hospital, under the matronship of Miss Tice, who was recently appointed to that post, the whole of the nursing staff have now one day off in seven. A number of interesting new appointments to the nursing staff of this hospital are announced on page 462 of this week's issue, including that of sister tutor, a post which is to be filled by Miss E. H. Gilbert, late assistant matron of Birmingham General Hospital.

---

The King held an Investiture in the Ball Room of Buckingham Palace at 10.30 on February 14. The following ladies were personally decorated by His Majesty with the Order of the Royal Red Cross (First Class).—*Q.A.I.M.N.S.* (*India*): Matron Irene McNally; *Q.A.I.M.N.S.R.*: Sister Mildred Wells; *Canadian A.N.S.*: Sister Lilian Pidgeon; *Australian A.N.S.*: Matron Edith Cornwell, Sister Alma Bennett, Sister Josephine Dunn, and Sister Julia Hart. The Royal Red Cross (Second Class) was bestowed by the King on Sister Isabella Jobson, Sister Leah Rosenthal, and

**ROUND THE HOSPITALS—(continued).**

Matron Annie Walker. *Voluntary Aid Detachment*: The Lady Elizabeth Keppel, Miss Cecilia Lawley, the Hon. Ursula Lawley, and Miss Lorna Leatham. *Canadian Army Nursing Service*: Sister Edna Auger, Sister Harriett Jukes, and Sister Gertrude Reid. *Australian Army Nursing Service*: Sister Helen Homan, Sister Elizabeth Horne, Sister Lily Mackenzie, and Sister Constance Stone. *New Zealand Army Nursing Service*: Sister Maud Atkinson and Sister Blanch Huddleston. At the Investiture held on the following day the King conferred the Order of the Royal Red Cross, First Class, on Matron Janet Macdonald, Can. A.N.S., and the Second Class of the Order on Staff Nurse Evelyn Walton, Q.A.I.M.N.S.(R.). Her Majesty Queen Alexandra received the recipients of the Order subsequent to the Investiture.

---

A DEFINITE proposal has been made by the medical officer of health for Greenwich for the partial training of suitable persons in infant welfare centres to act as home helps. The Council proposes to pay a salary to these helps and estimates the cost at £80 per annum each—board and lodging £40, wages £30, uniform £10. It seems a thousand pities not to pay a little more and train the women more efficiently. There is grave danger in utilising women who have had but a few weeks' or months' experience in maternity centres, and crèches, for by this means a door is opened for the admission of the partly-trained into the profession. Nothing demands more careful watching at the present moment than the facilities offered for sham training by municipal welfare enterprises.

---

THE Infirmary Committee at Newcastle-upon-Tyne have recommended an eight-hour day, exclusive of Sundays, for the nurses, and their scheme has been approved by the Guardians. The first relay take from 6 A.M. to 2 P.M.; the second relay from 2 P.M. to 10 P.M.; the third relay 10 P.M. to 6 A.M. The matron will arrange for a proportion of the nurses to be on leave from Saturday afternoon to Monday afternoon. The sisters' hours have been left for further consideration. It was stated by some of the Committee that the scheme would not involve any great addition to the nursing staff, but the staff would have to be kept up to its maximum. The new arrangement does honour to the Board.

---

IN recognition of the services rendered in connection with the nursing of the patients in the temporary influenza hospital opened during the epidemic, the Leeds Corporation Health Committee has voted gratuities to members of the Infant Welfare Staff as follows:—Miss M. Carter £5, and the Misses M. Belcher, F. Wese, B. Bolton £2 each.

---

STRIKES among male and female attendants at Irish asylums are gaining ground and are likely to be imitated. In Limerick Asylum, which contains 600 inmates, no one is left in charge. Some of the inmates have escaped and the relatives are being asked to remove the others. The Committee have attempted to come to an understanding with their employees on the basis of a 56-hour week with time-and-a-half to be allowed for over-work, but things have gone too far, and the proposal has been refused. The 56-hour week is already in working order at Clonmel Asylum, and had it been conceded to Limerick prior to the strike would no doubt have satisfied the overworked attendants. At Mullingar Asylum 140 male and female attendants have come out on strike and are marching in procession through sympathetic crowds in the town. They demand an extra £1 a week to meet war conditions. In this asylum 1,000 patients are left practically without attendance, but the strikers have left four nurses and four male attendants on duty for serious cases. Hardly any class of workers has been less studied in the past than the male and female staffs in asylums. If their long patience has at length broken down we must hesitate to blame unreservedly. The policy which has led to this deplorable extremity needs rapid overhauling in every asylum in the kingdom.

---

NURSING scholarships to the value of £200 are being given by the East Riding County Council in order to attract women of good attainments to the nursing service of the county. Those who obtain these scholarships will be expected to enter into an undertaking to work in the East Riding for three years at least after the completion of their training.

---

A TOUCHING little ceremony took place at the Infirmary, Ore, a few days ago when the Chaplain, nursing staff and officers assembled to bid good-bye to Miss Okey on her departure after twenty-four years' service as superintendent nurse. Mrs. Mills, the oldest patient, whose residence dates thirty years back, presented a charming dressing-gown and slippers from the patients, and she and other patients expressed their gratitude for all that Nurse Okey had done to make them happy. They were then to the number of 280 regaled by Nurse Okey with a "pre-war" high tea. A second presentation was made by Dr. Culhane, the medical officer, on behalf of Nurse Okey's numerous friends. It consisted of a gold wristlet-watch with an album, and was made the occasion of conveying to the late superintendent how warmly her work among the sick poor was appreciated by those best qualified to judge.

---

WE find in the January number of the American *Journal of Nursing*, under the name of Miss Grace Allison, Chief Nurse, No. 4 Base Hospital, some interesting and very cordial references to the reception accorded American war nurses on their arrival in this country. "We arrived in London on May 19. As this was the first appearance of America's entrance into the war England was most solicitous as to our reception. The Dowager Countess of Airlie, representing Queen Alexandra,

**ROUND THE HOSPITALS—(*continued*).**

and Lady Roberts, Miss Becher (the British Matron-in-Chief), Miss Lloyd Still (Matron of St. Thomas's Hospital), Miss Cox-Davies, and others were there to welcome us. As I look back at this experience I wonder whether the United States would have shown the same honour to the nursing profession as did England in receiving nurses officially at the War Department?"

From the other side of the Atlantic comes cordial endorsement of the proposal to secure the term "Nursing Sister" as the legal style of address for all registered nurses. "Had the nurses of America at the inception of registration adopted a new and distinct term of their own to indicate nurses who were considered fully qualified instead of endeavouring to monoplise the use of an old familiar word, dating back to ancient days, which the public had used in a general way without hindrance, a great deal of opposition would have been avoided and much precious time and energy conserved." This from the "Trained Nurse and Hospital Review" of New York. We trust the use of the term "Nursing Sister" may be secured for all nurses whose names are on the Register. A stroke of the pen would do it. In Clause 3 of College Bill the words "It shall be the duty of the General Nursing Council to form and to keep a General Register of Nurses" would need alteration to "a General Register of Nursing Sisters." In Clause 14 in place of "no person shall be entitled to take or use the name or title of Registered Nurse" the words "or that of Nursing Sister" would be added, the title of registered male nurse or registered mental nurse remaining unaffected. And the same alteration would have to be effected in other clauses. It is a matter of more importance than the lay mind can apprehend.

The members of Queen Mary's Needlework Guild met on February 1, in their stately quarters at Friary Court, St. James's Palace, for the last time. The Guild was formed within a week of the declaration of war, and those who have given their services have lately been awarded by Her Majesty a bar to their badge for each year's work. It is estimated that more than 14 million articles have passed through the packing departments. The Guild membership has passed its million, and it has 620 branches situated in every part of the world. One of the last consignments sent forth was fifteen tons of comforts for Russia. Nurses and hospital managers will learn with great satisfaction that the organisation which has produced such magnificent results is not to be entirely disbanded. It is premature to speculate on the manner in which this immense volume of good will evoked by the Guild could be turned to account in social reconstruction. But Her Majesty, who as an inspiring force has so brilliantly succeeded in organising the whole world to aid on a purely voluntary basis in the relief of misery, may be safely trusted with future developments.

The proposed French "Institute of Natality" is part of the movement in progress in many European countries for countering the falling birth-rate. Briefly it aims at making parenthood a profitable calling. Premiums are to be given to mothers and prospective mothers, the daily allowance suggested for the mother who does no work during the last four months of her pregnancy ranging from 1*s.* 10*d.* per day in villages to 4*s.* 2*d.* in the capital. To father and mother are to be granted a premium of £24 for each of the first two children, £40 for the third, £60 for the fourth, and thereafter £40 for each addition to the family. Where there are from six to eight legitimate children a pension of £20 per annum for life is granted to the parents in old age, rising to £80 for twelve legitimate children and over. The Bill is to be introduced into the Chamber by M. Lairolle.

The value of inspection from headquarters is illustrated by an incident which has recently been brought to our notice at the Camberwell Poor-Law Infirmary. An inspector from Whitehall found on inquiry that the nurses in the children's wards were not getting sufficient off-duty time in the course of the day to afford them proper exercise in the open-air. The result was an immediate decision on the part of the Guardians to give the children's nurses two hours off-duty daily. It may seem surprising that this was not done before. But the common practice of setting three nurses to do the work of six in the children's wards in Poor-Law institutions inevitably results in the sacrifice of the nurses.

Lady Rhondda, speaking at the Royal Institute of Public Health on "Women in the Ministry of Health," had some pregnant advice to offer. She urged the importance of getting women to help in initiating the policy they would be required to carry out, and expressed her pleasure at Dr. Addison's decision to have advisory councils by which the Minister could be kept in touch with public opinion. In matters of health women aided more than men to form public opinion, and it was women who could prevent new laws becoming dead letters. She would like to see a Council of women attached to the Health Ministry in addition to the representation of women on other councils within the Ministry. The importance of getting nurses as representatives of the different health services to sit on these councils cannot be too constantly kept in view. Until more nurses qualify themselves for taking part in initiating policy, their work must always fail of its highest public utility.

A complete maternity and child welfare scheme could be established in all the 28 boroughs of London at a cost of not more than 1d. rate. So Lord Downham estimates, and he, like Lady Rhondda, urges the importance of getting more women to sit on the Borough Council and the L.C.C.

